# Impact of gastroesophageal reflux on longitudinal lung function and quantitative computed tomography in the COPDGene cohort

**DOI:** 10.1186/s12931-020-01469-y

**Published:** 2020-08-03

**Authors:** Arianne K. Baldomero, Chris H. Wendt, Ashley Petersen, Nathaniel T. Gaeckle, MeiLan K. Han, Ken M. Kunisaki, for the COPDGene Investigators

**Affiliations:** 1 Division of Pulmonary, Allergy, Critical Care, and Sleep Medicine, Minneapolis VA Health Care System, One Veterans Drive, Mailstop: Pulmonary 111N, Minneapolis, MN 55417 USA; 2 Division of Pulmonary, Allergy, Critical Care, and Sleep Medicine, University of Minnesota, Minneapolis, MN USA; 3 Division of Biostatistics, University of Minnesota, Minneapolis, MN USA; 4 Division of Pulmonary and Critical Care Medicine, University of Michigan, Ann Arbor, MI USA

**Keywords:** Pulmonary disease, Chronic obstructive, Gastroesophageal reflux, Respiratory function tests, Spirometry, Longitudinal study, Computed tomography

## Abstract

**Rationale:**

Gastroesophageal reflux disease (GERD) is a common comorbidity in chronic obstructive pulmonary disease (COPD) and has been associated with increased risk of acute exacerbations, hospitalization, emergency room visits, costs, and quality-of-life impairment. However, it remains unclear whether GERD contributes to the progression of COPD as measured by lung function or computed tomography.

**Objective:**

To determine the impact of GERD on longitudinal changes in lung function and radiographic lung disease in the COPDGene cohort.

**Methods:**

We evaluated 5728 participants in the COPDGene cohort who completed Phase I (baseline) and Phase II (5-year follow-up) visits. GERD status was based on participant-reported physician diagnoses. We evaluated associations between GERD and annualized changes in lung function [forced expired volume in 1 s (FEV_1_) and forced vital capacity (FVC)] and quantitative computed tomography (QCT) metrics of airway disease and emphysema using multivariable regression models. These associations were further evaluated in the setting of GERD treatment with proton-pump inhibitors (PPI) and/or histamine-receptor 2 blockers (H_2_ blockers).

**Results:**

GERD was reported by 2101 (36.7%) participants at either Phase I and/or Phase II. GERD was not associated with significant differences in slopes of FEV_1_ (difference of − 2.53 mL/year; 95% confidence interval (CI), − 5.43 to 0.37) or FVC (difference of − 3.05 mL/year; 95% CI, − 7.29 to 1.19), but the odds of rapid FEV_1_ decline of ≥40 mL/year was higher in those with GERD (adjusted odds ratio (OR) 1.20; 95%CI, 1.07 to 1.35). Participants with GERD had increased progression of QCT-measured air trapping (0.159%/year; 95% CI, 0.054 to 0.264), but not other QCT metrics such as airway wall area/thickness or emphysema. Among those with GERD, use of PPI and/or H_2_ blockers was associated with faster decline in FEV_1_ (difference of − 6.61 mL/year; 95% CI, − 11.9 to − 1.36) and FVC (difference of − 9.26 mL/year; 95% CI, − 17.2 to − 1.28).

**Conclusions:**

GERD was associated with faster COPD disease progression as measured by rapid FEV_1_ decline and QCT-measured air trapping, but not by slopes of lung function. The magnitude of the differences was clinically small, but given the high prevalence of GERD, further investigation is warranted to understand the potential disease-modifying role of GERD in COPD pathogenesis and progression.

**Clinical trials registration:**

NCT00608764.

## Introduction

Gastroesophageal reflux disease (GERD) is a common comorbidity in chronic obstructive pulmonary disease (COPD). The prevalence of self-reported GERD in those with COPD is reported to be between 17% [1] to 54% [2] while studies using 24-h pH probe and manometry report prevalence as high as 78% [3]. Pulmonary microaspiration and vagal-mediated reflex bronchoconstriction have been proposed as possible mechanisms by which GERD contributes to COPD outcomes [4, 5]. Cross-sectional studies have consistently shown that, compared to COPD patients without GERD, those with GERD experience more frequent acute exacerbations [6–9], more hospitalizations and emergency room visits [2, 10–14], higher healthcare costs [15], and worse quality-of-life [12, 16]. However, it remains unclear whether GERD contributes to COPD pathogenesis and progression as measured by lung function or quantitative computed tomography (QCT) [17].

Cross-sectional studies that evaluated the relationship between GERD and lung function have revealed conflicting results – some studies observed worse airflow obstruction [2, 18, 19] in those with GERD, while other studies showed no significant relationship between GERD and lung function [12, 20–22]. Due to the cross-sectional design of these studies, we cannot derive definitive conclusions about the causal associations between GERD and COPD disease progression. Therefore, to evaluate if GERD is associated with COPD disease progression as measured by lung function or quantitative chest imaging, we analyzed the data from a large, longitudinal, multicenter cohort study. To our knowledge, this is the first study to longitudinally assess both lung function and quantitative chest imaging over a five-year period to evaluate the association between GERD and COPD disease progression.

## Methods

### Patient selection

COPDGene (ClinicalTrials.gov Registration # NCT00608764) is an ongoing multicenter, longitudinal study designed to investigate the genetic and epidemiologic characteristics of smoking-related lung disease. A complete description of the protocol has been published previously [18]. Briefly, the primary inclusion criteria are: self-identified racial/ethnic category of non-Hispanic white or African-American, 45–80 years old, with a minimum of 10 pack-year smoking history (except for a small number of non-smoking controls). For the current analysis, we selected from the full cohort of 10,720 enrolled participants and included participants who were former or current smokers and completed both baseline (Phase I, 2008–2011) and 5-year follow-up (Phase II, 2012–2016) study visits. The research protocol was approved by the institutional review board at each participating institution and all participants provided written informed consent.

### Diagnosis of GERD

Our primary predictor variable was the presence or absence of GERD. Standardized medical history and medication inventories were administered by research staff. GERD diagnosis was assessed by asking participants, “Have you ever been told by a physician that you have gastroesophageal reflux?” [16, 19]. To obtain an accurate medication list, participants were instructed to bring all current medications to the study visit. All medications were captured on the medications questionnaire, including GERD-related medications such as proton-pump inhibitors (PPI) and histamine receptor-2 blockers (H2 blockers).

### Lung function

Our primary outcome variable was rate of lung function decline, as assessed by post-bronchodilator spirometry. Participants underwent spirometry (EasyOne™ spirometer; ndd, Andover, MA) before and after administration of 180 μg of albuterol (via Aerochamber Activis, Parsippany, NJ) at Phase I and Phase II. Percent predicted and lower limit of normal (LLN) values were obtained using National Health and Nutrition Examination III reference equations for spirometry [20]. COPD severity was assessed using spirometry criteria outlined by the Global Initiative for Obstructive Lung Disease (GOLD) guidelines [21]. GOLD 0, defined as forced expiratory volume in 1 s/forced vital capacity (FEV_1_/FVC) ≥0.7 and an FEV_1_ < 80% predicted in current or former smokers without COPD, is not currently included in the GOLD guidelines, but was used previously [22]. Preserved ratio and impaired spirometry (PRISm) was defined as a post-bronchodilator FEV_1_/FVC ≥0.7 and an FEV_1_ > 80% predicted in former or current smokers [23, 24]. Rates of FEV_1_ and FVC changes per year were calculated by dividing the differences between Phase I and Phase II by the number of years between visits.

### Quantitative computed tomography (QCT) imaging measurements

Our secondary outcomes were QCT-based measures of lung disease. Participants included in this analysis had high-resolution CT scans at full inspiration at Phase I and Phase II to assess emphysema and airway disease. Quantitative imaging analysis were performed using VIDA (VIDA Diagnostics, Iowa City, IA; http://www.vidadiagnostics.com) software and Thirona (https://thirona.eu/) software. QCT outcomes included airway wall thickness (AWT)-Pi10 (square root of the wall area of a theoretical airway of 10 mm luminal perimeter), airway wall area (100 X wall area/total bronchial area), air trapping (percent of lung with attenuation values less than − 856 HU on expiratory CT) [25], emphysema (percent of voxels on inspiratory CT with attenuation values less than − 950 Hounsfield Units (HU), and Perc15 lung density (the 15th percentile point defined as the HU below which the 15% of voxels with the lowest density are distributed, adjusted for CT-based lung volumes) [26]. Rates of QCT imaging measurement changes per year were calculated by dividing the differences between Phase I and Phase II by the number of years between visits.

### Statistical analysis

Demographic, clinical, and lung health characteristics were compared between those with and without GERD using descriptive statistics. GERD was assessed identically at the Phase I and Phase II visits, and in our primary analysis, we categorized those with GERD as reporting GERD at either visit and compared outcomes to those with no GERD at either visit. Associations between GERD and longitudinal changes in spirometry and QCT chest measurements were assessed using linear regression models with sandwich standard errors. We present the outcome data using three models: Model 1 is unadjusted; Model 2 covariates included age, sex, race, whether the patient smoked between Phase I and Phase II, body mass index (BMI), clinical center, and FEV_1_% predicted at Phase I; and Model 3 included covariates in Model 2 and whether or not the patient had ≥1 acute exacerbation of COPD between Phase I and Phase II.

Secondary analyses compared changes in spirometry between [1] those with ‘persistent GERD’ (GERD at both Phase I and Phase II) vs. no GERD at either visit, [2] those with ‘incident GERD’ (no GERD at Phase I, but GERD at Phase II) vs. no GERD, and [3] those with ‘resolved GERD’ (GERD at Phase I, but not Phase II) vs. no GERD. Adjustments were made for the same covariates as Model 3 in the primary analyses. Additionally, logistic regression models were used to estimate the odds of having rapid FEV_1_ decline (defined as FEV_1_ decline of ≥40 mL/year) for those with vs. without GERD after adjustment for the same covariates. Lastly, we explored associations between GERD treatment (PPI and/or H_2_ blocker at Phase I and/or Phase II) and changes in lung function using linear regression models.

## Results

Data were available for 5728/10,720 (53.4%) participants who were former or current smokers and completed both Phase I (baseline, 2008–2011) and Phase II (5-year follow-up, 2012–2016) study visits (Supplemental Fig. 1S). Physician-diagnosed GERD was reported in 2101/5728 (36.7%) participants at either Phase I and/or Phase II (Table 1). Those with GERD were more frequently female (55.6% vs. 45.9%), GOLD stages ≥2 (38.0% vs. 28.3%), and prescribed inhaled therapies (43.5% vs. 26.1%). In addition, participants with GERD were more likely to have experienced acute exacerbations of COPD (26.8% vs. 13.8%) and severe exacerbations of COPD (12.1% vs. 6.9%), worse SGRQ total score (median of 25 vs. 14), and more dyspnea as assessed by mMRC score ≥ 2 (45.3% vs. 30.0%). Cohort characteristics at Phase I comparing those with (*n* = 4031) and without (*n* = 1697) available QCT measurements at both visits are provided in the Supplemental Table 1S.

**Table 1:** Cohort characteristics at Phase I by gastroesophageal reflux (GERD) status. Data are presented as mean (standard deviation), median (first quartile, third quartile), or n (%)

	Phase I	
	No GERD	GERD
	*n* = 3627	*n* = 2101
**Demographics**		
Age, years	59.0 (8.74)	60.9 (8.38)
Female	1668 (46%)	1168 (56%)
African American	1269 (35%)	489 (23%)
Current smoker	1928 (53%)	862 (41%)
Pack-years	40.9 (22.2)	45.3 (25.4)
BMI, kg/m^2^	28.6 (6.0)	29.9 (6.3)
Education beyond HS	2348 (65%)	1396 (66%)
**Spirometry**		
FEV_1_% predicted	82 (23)	77 (23)
FVC % predicted	90 (17)	87 (17)
FEV_1_/FVC	0.70 (0.14)	0.67 (0.15)
**GOLD stage**		
PRISm	438 (12%)	263 (13%)
GOLD 0	1826 (51%)	851 (41%)
GOLD 1	321 (8.9%)	182 (8.7%)
GOLD 2	626 (17%)	483 (23%)
GOLD 3	309 (8.6%)	258 (12%)
GOLD 4	86 (2.4%)	55 (2.6%)
**Inhaled therapies**		
SABA	776 (22%)	763 (37%)
LABA	57 (1.6%)	85 (4.1%)
ICS	137 (3.9%)	144 (7.0%)
ICS/LABA	438 (12%)	460 (22%)
LAMA	362 (10%)	389 (19%)
**Quantitative CT chest**		
AWT-Pi10, mm	2.2 (0.58)	2.3 (0.58)
Airway wall area, %	50 (8.3)	50 (8.2)
Air trapping, %	19 (17)	22 (18)
Emphysema, %	5.1 (8)	6.4 (9.1)
Perc15 lung density, HU	78 (22)	76 (23)
**Clinical outcomes**		
Acute exacerbation	499 (14%)	563 (27%)
Severe exacerbation	252 (6.9%)	254 (12%)
Cough	1126 (31%)	815 (39%)
Phlegm	1126 (31%)	803 (38%)
Wheeze	1380 (38%)	1093 (52%)
SGRQ, total score	14 (3.8, 32)	25 (9.3, 45)
mMRC ≥2	1086 (30%)	949 (45%)
6-MWD, feet	1448 (368.8)	1369 (370.4)

*Abbreviations: GERD* gastroesophageal reflux disease, *BMI* body mass index, *HS* high school, *FEV*_*1*_ forced expiratory volume in 1 s, *FVC* forced vital capacity, *PRISm* preserved ratio impaired spirometry, *GOLD* Global Initiative for Chronic Obstructive Lung Disease, *SABA* short-acting beta-agonist, *LABA* long-acting beta-agonist, *ICS* inhaled corticosteroid, *ICS/LABA* combination inhaled corticosteroid and long-acting beta-agonist, *LAMA* long-acting muscarinic agonist, *TLC* total lung capacity, *AWT-Pi10* airway wall thickness at an internal perimeter of 10 mm, *HU* Hounsfield Units, *SGRQ* St. George’s Respiratory Questionnaire, *mMRC* modified Medical Research Council Dyspnea Scale and *6-MWT* 6-min walk distance

Compared to participants without GERD, participants with GERD at Phase I and/or Phase II had faster decline in FEV_1_ (difference of − 3.64 mL/year, 95% confidence interval (CI), − 6.56 to − 0.73) and FVC (difference of − 4.26 mL/year; 95% CI, − 8.52 to − 0.004), after adjustment for age, sex, race, smoking, BMI, clinic center, and FEV_1_% predicted (Table 2). When additionally adjusted for acute exacerbations, the estimates were attenuated and no longer statistically significant, with 95% confidence interval bounds crossing zero. Participants with GERD showed faster progression of air trapping (difference of 0.159%/year; 95% CI, 0.054–0.264) on QCT. We observed no association between the rate of change of AWT-Pi10 (μm/year), airway wall area (%/year), emphysema (%/year), Perc15 lung density (HU/year), and GERD status.

**Table 2:** Linear regression models of the association between gastroesophageal reflux disease (GERD) and slopes of lung function and Quantitative CT measures of lung disease. ß coefficients reflect the mean differences in the row outcome of interest between those with GERD compared to those without GERD

	Model 1: Crude	Model 2: Age, sex, race, smoked between Phase I and II, BMI, clinical center, and FEV_**1**_% predicted at Phase I	Model 3: Model 2 + Acute exacerbations ≥ 1 between Phase I and Phase II
	Unadjusted ß Estimate (95% CI)	Adjusted ß Estimate (95% CI)	Adjusted ß Estimate (95% CI)
**Spirometry**			
FEV_1_ (mL/year)	−0.01 (−2.92, 2.89)	**−3.64 (−6.56, − 0.73)**	−2.53 (−5.43, 0.37)
FVC (mL/year)	−2.76 (− 6.93, 1.42)	**−4.26 (−8.52, − 0.004)**	−3.05 (− 7.29, 1.19)
**Quantitative CT chest**			
AWT-Pi10 (μm/year)	2.31 (−1.86, 6.48)	1.28 (− 2.91, 5.48)	0.64 (−3.57, 4.84)
Airway wall area (%/year)	0.037 (− 0.022, 0.097)	0.013 (− 0.047, 0.073)	0.003 (− 0.058, 0.063)
Air trapping (%/year)	**0.117 (0.006, 0.227)**	**0.167 (0.063, 0.271)**	**0.159 (0.054, 0.264)**
Emphysema (%/year)	0.035 (− 0.010, 0.081)	0.015 (− 0.025, 0.055)	0.012 (− 0.028, 0.052)
Perc15 lung density (HU/year)	− 0.073 (− 0.207, 0.061)	− 0.024 (− 0.137, 0.089)	−0.023 (− 0.137, 0.090)

*Abbreviations: CT* computed tomography, *GERD* gastroesophageal reflux disease, *FEV*_*1*_ forced expiratory volume in 1 s, *FVC* forced vital capacity, *AWT-Pi10* airway wall thickness at an internal perimeter of 10 mm and *CI* confidence interval

In secondary analyses, ‘incident GERD’ (Phase I = ‘no’, Phase II = ‘yes’) was associated with faster decline in FEV_1_ and FVC compared to ‘any GERD’ (Phase I = ‘yes’ and/or Phase II = ‘yes’), ‘persistent GERD’ (Phase I = ‘yes’ *and* Phase II = ‘yes’), and ‘resolved GERD’ (Phase I = ‘yes’ and Phase II = ‘no’) (Table 3). The odds of rapid FEV_1_ decline (*n* = 2572) was significantly increased for those reporting ‘any GERD’ (adjusted odds ratio [aOR]: 1.20; 95% CI, 1.07 to 1.35), ‘persistent GERD’ (aOR: 1.23; 95% CI, 1.06 to 1.43), and ‘incident GERD’ (aOR: 1.33; 95% CI, 1.11 to 1.60); the only participants that did not have an increased odds of rapid decline was the ‘resolved GERD’ (aOR: 1.01; 95% CI, 0.82 to 1.26) group (Table 4).

**Table 3:** Multivariable linear regression models of the association between gastroesophageal reflux disease (GERD) and slopes of lung function. ß coefficients reflect the mean differences in the row outcome of interest between those with GERD compared to those without GERD

	Any GERD *n* = 2101	Persistent GERD *n* = 1080	Incident GERD *n* = 604	Resolved GERD *n* = 417
	Adjusted ß Estimate (95% CI)	Adjusted ß Estimate (95% CI)	Adjusted ß Estimate (95% CI)	Adjusted ß Estimate (95% CI)
**Spirometry**				
FEV_1_ (mL/yr)	−2.53 (− 5.43, 0.37)	− 2.07 (− 5.75, 1.61)	**−5.47 (− 9.87, − 1.07)**	−0.24 (− 5.87, 5.39)
FVC (mL/yr)	−3.05 (− 7.29, 1.19)	− 1.69 (− 7.01, 3.63)	−6.29 (− 13.0, 0.42)	−1.62 (− 9.38, 6.15)

Adjustment was made for the following variables: age, sex, race, smoked between phase I and II, BMI, clinical center, FEV_1_% predicted at Phase I, and acute exacerbation ≥1 between phase I and II

*Definitions:* Any GERD, Phase I = ‘yes’ or Phase II = ‘yes’; Persistent GERD, Phase I = ‘yes’ and Phase II = ‘yes’; Incident GERD, Phase I = ‘no’ and Phase II = ‘yes’; and Resolved GERD, Phase I = ‘yes’ and Phase II = ‘no’

*Abbreviations: GERD* gastroesophageal reflux disease, *FEV*_*1*_ forced expiratory volume in 1 s, *FVC* forced vital capacity and *CI* confidence interval

**Table 4:** Multivariable logistic regression models of the association between gastroesophageal reflux disease (GERD) and rapid FEV_1_ decline (FEV_1_ decline of ≥40 mL/year, *n* = 2572). Adjusted odds ratios reflect the relative odds of rapid FEV_1_ decline between those with GERD, compared to those without GERD

	Adjusted Odds Ratio (95% CI)
Any GERD (*n =* 2101)	**1.20 (1.07, 1.35)**
Persistent GERD (*n =* 1080)	**1.23 (1.06, 1.43)**
Incident GERD (*n =* 604)	**1.33 (1.11, 1.60)**
Resolved GERD (*n =* 417)	1.01 (0.82, 1.26)

Adjustment was made for the following variables: age, sex, race, smoked between phase I and II, BMI, clinical center, FEV_1_% predicted at Phase I, and acute exacerbation ≥1 between phase I and II

*Definitions:* Any GERD, Phase I = ‘yes’ or Phase II = ‘yes’; Persistent GERD, Phase I = ‘yes’ and Phase II = ‘yes’; Incident GERD, Phase I = ‘no’ and Phase II = ‘yes’; and Resolved GERD, Phase I = ‘yes’ and Phase II = ‘no’

*Abbreviations: GERD* gastroesophageal reflux disease, *FEV*_*1*_ forced expiratory volume in 1 s and *CI* confidence interval

Among our 5728 study participants, pharmacologic treatment with PPIs was reported by 990 (24%) and H_2_ blockers by 260 (6.5%), of whom most (81%) reported GERD at either visit, though 19% did not report GERD at either visit. Among those with GERD, treatment with PPI and/or H_2_ blocker at either Phase I and/or Phase II was associated with faster decline in lung function (Table 5). Among participants with GERD, the decline in both FEV_1_ (difference of − 6.61; 95% CI, − 11.9 to − 1.36) and FVC (difference of − 9.26; 95% CI, − 17.2 to − 1.28) were faster in those receiving PPI and/or H2 blocker compared to those who are not receiving either medication. Among those without GERD, PPI and/or H_2_ blocker treatment was not associated with lung function decline, though these estimates had less precision than in those with GERD due to the smaller sample.

**Table 5:** Multivariable linear regression models of the association between treatment with proton-pump inhibitor (PPI) and/or H_2_ blocker and slopes of lung function, in those with and without gastroesophageal reflux disease (GERD). ß coefficients reflect the mean differences in the row outcome of interest between those with treatment with PPI and/or H_2_ blocker, compared to those not receiving treatment

	Adjusted ß Estimate (95% CI)
GERD (n = 960)	
FEV_1_ (mL/year)	**−6.61 (−11.9, − 1.36)**
FVC (mL/year)	**−9.26 (− 17.2, − 1.28)**
No GERD (n = 221)	
FEV_1_ (mL/year)	6.38 (−3.04, 15.8)
FVC (mL/year)	3.97 (−7.66, 15.6)

Adjustment was made for the following variables: age, sex, race, smoked between phase I and II, BMI, clinical center, FEV_1_% predicted at Phase I, and acute exacerbation ≥1 between phase I and II

PPI include esomeprazole, lansoprazole, pantoprazole, omeprazole, rabeprazole

H_2_ blocker include cimetidine, ranitidine, famotidine, nizatidine

PPI and/or H_2_ blocker (59.8%), PPI (52.4%), H_2_ blocker (13.0%)

*Abbreviations: GERD* gastroesophageal reflux disease, *FEV*_*1*_ forced expiratory volume in 1 s, *FVC* forced vital capacity. *PPI* proton pump inhibitor, *H*_*2*_*blocker* histamine receptor-2 blocker and *CI* confidence interval

No significant differences in the slopes of change of the QCT chest measures was found in those taking PPI and/or H_2_ blockers compared to those who were not taking medications (Supplemental Table 2S).

## Discussion

Data from our large, multicenter, longitudinal cohort suggest that GERD may contribute to progressive loss of lung function and increases in air trapping over time. Although other studies have found cross-sectional associations between GERD and lung health, to our knowledge, our study is the first to use a longitudinal study design over a five-year period and assess both lung function and quantitative imaging measurements.

Despite several statistically significant associations between GERD and longitudinal changes in spirometry and QCT measures, we note that the magnitude of the effect sizes were clinically small, with point estimates of 2–5 mL/year faster FEV_1_ decline among those with GERD. Although this degree of faster lung function decline might not be expected to lead to significant clinical problems, we note that the effect size of cigarette smoking has been estimated at 4–27 mL/year faster decline, so an additional 2–5 mL/year might still contribute to disease progression, in combination with other factors that might contribute to lung function decline such as non-cigarette smoke exposures, respiratory infections, and abnormal inflammatory responses [27, 28]. Although we have little data to guide us in categorizing more significant rates of QCT changes over time, for spirometry, we applied a common definition of rapid FEV_1_ decline of ≥40 mL/year [29]. In this categorical logistic regression analysis, we saw that GERD was associated with a 20–33% increased odds of rapid decline. Although we must advise caution in interpreting this secondary analysis, these results suggest there might be a subgroup of persons more susceptible to pulmonary effects of GERD, but further research is needed to explore this hypothesis.

We included acute exacerbations as a covariate in our models as episodes of acute exacerbations have been known to accelerate lung function decline [30]. We found that rapid FEV_1_ decline and progression in QCT-measured air trapping were associated with GERD, but the estimates of slopes of FEV_1_ and FVC were attenuated compared to the model that did not include acute exacerbations as a covariate.

We evaluated loss of lung function both as continuous variables (mL/year) and as a categorical variable (FEV_1_ decline ≥40 mL, yes vs. no), then stratified GERD into ‘persistent’, ‘incident’, and ‘resolved.’ Rapid decline in FEV_1_ is a strong predictor of mortality and COPD-related hospitalization [31]. This current study suggests that GERD is an independent predictor of rapid FEV_1_ decline, using a multivariate logistic regression model controlling for age, sex, race, smoking status, BMI, FEV_1_% predicted at baseline, and acute exacerbations. Participants with ‘resolved GERD’ do not appear to have increased odds of rapid FEV_1_ decline raising the question about the potential role of GERD treatment in slowing lung function decline, which will need to be addressed in future clinical trials.

Smaller cross-sectional studies that have evaluated the relationship between lung function severity and GERD have shown mixed results. Mokhlesi et al. [32] found that symptomatic GERD was more prevalent in COPD patients with FEV_1_ ≤ 50% compared to those with FEV_1_ > 50% (23% vs. 9%, respectively; *p* = 0.08), while Rogha et al. [2] showed that patients with GERD have more severe COPD compared to those without GERD (GOLD stage ≥2 or higher: 88% vs. 67%, respectively; *p* = 0.005) supporting our findings. In contrast, several other studies found no association between lung function and GERD, possibly due to relatively small sample sizes [12, 33–35]. Our present study expands the literature on the relationship between lung function and GERD by adding temporal dimension and a larger sample size.

In addition to adding a temporal dimension in the assessment of the impact of GERD in lung function, we also evaluated whether GERD contributes to the progression of small airway disease and emphysema over time using QCT. Small airway obstruction and emphysematous lung destruction reflect abnormalities in lung function [36]. Airway changes using QCT in the context of aspiration have been evaluated previously [37–42], but these studies are also limited to small cross-sectional studies. Hiller et al. found that patients with recurrent aspiration of gastric contents had QCT evidence of bronchial wall thickening (95%) and air trapping (44%) [42]. Similarly, Cardasis et al. found increased airway wall thickening on QCT of patients with pathologically-confirmed chronic occult aspiration of whom 96% had diagnosis of GERD [41]. We found that the rate of air trapping progression over 5 years was faster in those with GERD compared to those without GERD. This could represent the development of distal small airway disease as a result of ongoing pulmonary micro-aspiration of refluxed gastric material and/or vagally-mediated reflex bronchoconstriction in GERD [4, 5]. However, the slopes of the other QCT measurements of small airway disease (AWT-Pi10 and airway wall area) and emphysema (% emphysema and Perc15 lung density) were not different between those with and without GERD. We hypothesize that air trapping in the setting of GERD is a possible early measurable imaging manifestation of small airway disease, possibly an imaging finding that can be seen prior to the other QCT measurements of small airway disease and emphysema. This hypothesis will need to be addressed in future longitudinal studies.

The association between GERD and lung health bring into question the role of anti-reflux treatment in management of COPD. We observed that pharmacologic treatment with PPI and/or H_2_ blocker among participants with GERD was associated with an accelerated decline in FEV_1_ and FVC. Using the same COPDGene cohort, Martinez et al. showed that the use of PPI was associated with improved SGRQ total score, but also increased exacerbations highlighting the possibility of confounding-by-indication [16]. The significant decline in lung function with PPI and/or H_2_ blocker use in this cohort is also likely due to confounding-by-indication. Xiong et al. suggested that treatment of GERD with PPI in patients with COPD is associated with delayed deterioration of FEV_1_ after 1-year follow-up [43]. However, several other studies evaluating the efficacy of anti-reflux medications in COPD did not report on the impact of these pharmacologic therapies on lung function [12, 32, 44–46]. A limitation to the pharmacologic treatment of GERD is that anti-reflux medications do not target nonacid reflux and weakly acidic reflux. Surgical intervention with fundoplication, on the contrary, impacts both acid and non-acid reflux. Most of the literature on anti-reflux surgery focuses on lung transplant. Although the evidence is conflicting, a systematic review by Robertson et al. suggested that anti-reflux surgery provided benefit in lung function among lung transplant patients [47]. We did not find studies specifically addressing anti-reflux surgery in COPD and lung function. The effects of anti-reflux therapies in COPD outcomes, specifically lung function, are unclear highlighting the need for carefully-designed clinical trials.

Our study has limitations. GERD was based on self-report of a physician diagnosis, not on validated reflux questionnaires, pH monitoring, or esophageal manometry. Therefore, GERD misclassification might have affected our results. Because this is an observational study, we cannot establish causal inferences.

## Conclusion

GERD was associated with faster COPD disease progression as measured by rapid FEV_1_ decline and QCT-measured air trapping, but not by slopes of lung function. The magnitude of the differences was clinically small, but given the high prevalence of GERD, further investigation is warranted to understand the potential disease-modifying role of GERD in COPD pathogenesis and progression.

## Supplementary information

**Additional file: Supplemental Figure 1S.** Participant flow diagram. **Supplemental Table 1S.** Cohort characteristics at Phase I by availability of quantitative CT (QCT) measurements at both visits. **Supplemental Table 2S.** Multivariable linear regression models of the association between treatment with proton pump inhibitor (PPI) and/or H_2_ blocker (*n* = 960) and slopes of quantitative CT (QCT) measures of lung disease among those with gastroesophageal reflux disease (GERD).

## Data Availability

The datasets used are available from the corresponding author on reasonable request.
