# Supplementary material for: Impact of gastroesophageal reflux on longitudinal lung function and quantitative computed tomography in the COPDGene cohort
Source: Respir Res. 2020 Aug 3;21:203. doi: 10.1186/s12931-020-01469-y (PMC7397645; doi:10.1186/s12931-020-01469-y)
Supplement: Supplementary file 1 — Additional file: Supplemental Figure 1S. Participant flow diagram. Supplemental Table 1S. Cohort characteristics at Phase I by availability of quantitative CT (QCT) measurements at both visits. Supplemental Table 2S. Multivariable linear regression models of the association between treatment with proton pump inhibitor (PPI) and/or H2 blocker (n = 960) and slopes of quantitative CT (QCT) measures of lung disease among those with gastroesophageal reflux disease (GERD). [file 12931_2020_1469_MOESM1_ESM.docx]

**ONLINE DATA SUPPLEMENT**

**Supplemental Data for Article:**

**Impact of Gastroesophageal Reflux on Longitudinal**

**Lung Function and Quantitative Computed Tomography**

**in the COPDGene Cohort**

**Authors:**

Arianne K. Baldomero, MD, MS^1,2^, Chris H. Wendt, MD^1,2^, Ashley Petersen, PhD^3^ , Nathaniel T. Gaeckle, MD^2^, MeiLan K. Han, MD, MS^4^, Ken M. Kunisaki, MD, MS^1,2^, for the COPDGene Investigators

^1^Division of Pulmonary, Allergy, Critical Care, and Sleep Medicine, Minneapolis VA Health Care System, Minneapolis, MN

^2^Division of Pulmonary, Allergy, Critical Care, and Sleep Medicine, University of Minnesota, Minneapolis, MN

^3^Division of Biostatistics, University of Minnesota, Minneapolis, MN

^4^ Division of Pulmonary and Critical Care Medicine, University of Michigan, Ann Arbor, MI

**Contents of Online Data Supplement:**

**Supplemental Figure 1S.** Participant flow diagram

**Supplemental Table 1S.** Cohort characteristics at Phase I by availability of quantitative CT (QCT) measurements at both visits

**Supplemental Table 2S.** Multivariable linear regression models of the association between treatment with proton pump inhibitor (PPI) and/or H_2_ blocker (n=960) and slopes of quantitative CT (QCT) measures of lung disease among those with gastroesophageal reflux disease (GERD).

Enrolled in COPDGene Study

N = 10,720

Analysis Population

N = 5728

Missing GERD Status

N = 2

Never Smoker

N = 76

Smoker or Former Smoker

N = 5730

No Phase 1 Visit

N = 349

Onsite Phase 2 Visit

N = 5806

No Phase 2 Visit

N = 3962

At-Home Phase 2 Visit

N = 33

Limited Phase 2 Visit

N = 457

Phone Phase 2 Visit

N = 113

Completed Phase 1 Visit

N = 10,371

**Supplemental Figure 1S.** Participant flow diagram

**Supplemental Table 1S.** Cohort characteristics at Phase I by availability of quantitative CT (QCT) measurements at both visits

|  | Phase I | |  |
| --- | --- | --- | --- |
|  | **Available QCT** | **Missing QCT** | |
|  | n=4031 | n=1697 | |
| **Demographics** |  |  | |
| Age, years | 60.0 (8.64) | 59.1 (8.68) | |
| Female | 2003 (50%) | 833 (49%) | |
| African American | 1102 (27%) | 656 (39%) | |
| Current smoker | 1873 (46%) | 917 (54%) | |
| Pack-years | 42.4 (22.9) | 42.7 (24.8) | |
| BMI, kg/m^2^ | 29.0 (6.0) | 29.4 (6.5) | |
| Education beyond HS | 2708 (67%) | 1036 (61%) | |
| **Spirometry** |  |  | |
| FEV_1_ % predicted | 80 (23) | 79 (23) | |
| FVC % predicted | 89 (17) | 88 (17) | |
| FEV_1_/FVC | 0.69 (0.14) | 0.69 (0.14) | |
| **GOLD stage** |  |  | |
| PRISm | 465 (12%) | 236 (14%) | |
| GOLD 0 | 1896 (47%) | 781 (46%) | |
| GOLD 1 | 366 (9.1%) | 137 (8.1%) | |
| GOLD 2 | 787 (20%) | 322 (19%) | |
| GOLD 3 | 404 (10%) | 163 (9.7%) | |
| GOLD 4 | 95 (2.4%) | 46 (2.7%) | |
| **Inhaled therapies** |  |  | |
| SABA | 1023 (26%) | 516 (31%) | |
| LABA | 99 (2.5%) | 43 (2.6%) | |
| ICS | 196 (5.0%) | 85 (5.1%) | |
| ICS/LABA | 630 (16%) | 268 (16%) | |
| LAMA | 525 (13%) | 226 (14%) | |
| **Clinical outcomes** |  |  | |
| Acute exacerbation | 719 (18%) | 343 (20%) | |
| Severe exacerbation | 335 (8.3%) | 171 (10%) | |
| Cough | 1345 (33%) | 596 (35%) | |
| Phlegm | 1309 (32%) | 620 (37%) | |
| Wheeze | 1706 (43%) | 767 (45%) | |
| SGRQ, total score | 16 (5.0, 36) | 21 (6.5, 40) | |
| mMRC ≥2 | 1345 (33%) | 690 (41%) | |
| 6-MWD, feet | 1442 (366.5) | 1365 (377.3) | |

*Abbreviations:* GERD, gastroesophageal reflux disease; BMI, body mass index; HS, high school; FEV_1_, forced expiratory volume in 1 second; FVC, forced vital capacity; PRISm, preserved ratio impaired spirometry; GOLD, Global Initiative for Chronic Obstructive Lung Disease; SABA, short-acting beta-agonist; LABA, long-acting beta-agonist; ICS, inhaled corticosteroid; ICS/LABA, combination inhaled corticosteroid and long-acting beta-agonist; LAMA, long-acting muscarinic agonist; SGRQ, St. George’s Respiratory Questionnaire; mMRC, modified Medical Research Council Dyspnea Scale; and 6-MWT, 6-minute walk distance

Summaries shown are mean (standard deviation), n (percent), and median (first quartile, third quartile)

**Supplemental Table 2S.** Multivariable linear regression models of the association between treatment with proton pump inhibitor (PPI) and/or H_2_ blocker (n=960) and slopes of quantitative CT (QCT) measures of lung disease among those with gastroesophageal reflux disease (GERD). ß coefficients reflect the mean differences in the row outcome of interest between those with treatment with PPI and/or H_2_, compared to those not receiving treatment.

|  | Adjusted ß Estimate  (95% CI) |
| --- | --- |
| AWT-Pi10 (μm/year) | 4.68 (-2.79, 12.2) |
| Airway wall area (%/year) | 0.029 (-0.079, 0.136) |
| Air trapping (%/year) | 0.030 (-0.160, 0.220) |
| Emphysema (%/year) | -0.054 (-0.128, 0.020) |
| Perc15 lung density (HU/year) | 0.043 (-0.144, 0.229) |

Adjustment was made for the following variables: age, sex, race, smoked between phase I and II, BMI, clinical center, FEV_1_ % predicted at Phase I, and acute exacerbation ≥1 between phase I and II.

PPI include esomeprazole, lansoprazole, pantoprazole, omeprazole, rabeprazole

H2 blocker include cimetidine, ranitidine, famotidine, nizatidine.

PPI and/or H_2_ blocker (59.8%), PPI (52.4%), H_2_ blocker (13.0%)

*Abbreviations:* CT, computed tomography; GERD, gastroesophageal reflux disease; AWT-Pi10, airway wall thickness at an internal perimeter of 10 mm; HU, Hounsfield Units; PPI, proton pump inhibitor; H_2_ blocker, histamine receptor-2 blocker; and CI, confidence interval
